# Tumor-Derived Apoptotic Vesicles: With Death They Do Part

**DOI:** 10.3389/fimmu.2018.00957

**Published:** 2018-05-07

**Authors:** Morad-Remy Muhsin-Sharafaldine, Alexander D. McLellan

**Affiliations:** Department of Microbiology and Immunology, University of Otago, Dunedin, New Zealand

**Keywords:** extracellular vesicles, apoptotic vesicles, membrane blebs, chemotherapy, thrombosis

## Abstract

Tumor cells release lipid particles known as extracellular vesicles (EV) that contribute to cancer metastasis, to the immune response, and to thrombosis. When tumors are exposed to radiation or chemotherapy, apoptotic vesicles (ApoVs) are released in abundance as the plasma membrane delaminates from the cytoskeleton. Recent studies have suggested that ApoVs are distinct from the EVs released from living cells, such as exosomes or microvesicles. Depending on their treatment conditions, tumor-released ApoV have been suggested to either enhance or suppress anti-cancer immunity. In addition, tumor-derived ApoV possess procoagulant activity that could increase the thrombotic state in cancer patients undergoing chemotherapy or radiotherapy. Since ApoVs are one of the least appreciated type of EVs, we focus in this review on the distinctive characterization of tumor ApoVs and their proposed mechanistic effects on cancer immunity, coagulation, and metastasis.

## Introduction

The term extracellular vesicle (EV) describes membrane particles released from eukaryote cells, as well as prokaryote microorganisms (1). It is now evident that EV plays important roles in multiple biological systems involved in the control of homeostasis of the organism. For example, intercellular signaling is mediated by EV in processes, such as bone calcification, immune tolerance and activation, neuron-glia communication, wound repair, and hemostasis (2–7). Furthermore, EVs have been implicated in pathological conditions, such as viral and prion transfer, cardiovascular disease, thrombosis, autoimmune diseases (e.g., rheumatoid arthritis and multiple sclerosis), sickle cell anemia, and cancer (8–14).

Extracellular vesicle can be isolated *via* differential and/or density gradient centrifugation based on their relative size and density. Depending on their parental cell type and their cellular site of origin, EVs differ in terms of size, composition, density, and other biochemical and structural properties (15, 16). Exosomes, one of the smallest EV, are released from a large spectrum of living cells and range from 40–100 nm in diameter [isolated at sedimentation speeds of ≥100,000 × *g*; Ref. (17)]. While the differentiation or activation state of primary cells is critical for exosome release (18), Wolfers et al. have shown that murine and human tumor cell lines constitutively release exosomes (19). After their discovery by electron microscopy in 1981 (20), numerous studies have shown that exosomes may function as intercellular messengers in a diverse range of roles controlling cellular physiology and pathology (5, 19, 21–24).

Living cells also secrete larger, membrane-derived EV known as microvesicles (MV). These were first described by Chargaff and West in 1946 and were later characterized as a predominant procoagulant product of degranulating platelets (25, 26). MVs range in size (0.1–2 µm in diameter) and have been shown to be constitutively released by tumor cells potentially carrying oncogenes (27). In addition, “migratory benign cells-derived EV” structures, termed migrasomes (≤3 μm), that harbor internal EV, have also recently been described (28). Tumor-derived EV (100–500 nm) that transport epidermal growth factor receptor variant III, overexpressed in the parental human U373 astrocytoma tumor cell line have been referred to as “oncosomes” (29). Other studies that have used this term and verified the tumor origin of “oncosomes” due to other oncogenic cargo (30–33). However, it is still not clear from the original (29), or clarified definitions (34), if oncosomes contain “oncogenes” (i.e., transforming nucleic acids), “oncogenic receptors” (oncogene products; i.e., epidermal growth factor receptor variant III polypeptide), or whether this terminology merely reflect their functional transforming ability (transfer of oncogenic activity). Since the term oncosomes originates from a single cell line studied (29), the size range, cargo, or morphological features cannot yet be extrapolated to other tumor cells. Moreover, oncosomes have yet to be included in the ISEV guidelines (35, 36).

During apoptosis, cellular contents are packaged into apoptotic blebs (0.03–5 µm) for clearance with minimal perturbation/inflammation to the surrounding tissues (15, 37). The term “apoptotic bodies” usually refers to the larger (1–5 µm) bleb fraction (38, 39) that may contain a proportion of nuclear content and are released when the plasma membrane delaminates from the cytoskeleton (37). Due to their variable size range, apoptotic blebs are isolated at different sedimentation speeds [1,000–110,000 × *g*; Ref. (15, 40–43)]. An obvious problem with definition occurs when studies isolate smaller apoptotic blebs at high speeds (≥100,000 × *g*); resulting in likely contamination with exosomes due to the similar sedimentation forces used in the isolation of these two EV sub-types. However, contaminating exosomes will have distinguishable markers (discussed later) that could be utilized to enhance apoptotic vesicles (ApoVs) purity. In addition, there is no consensus regarding the nomenclature for the smaller fraction of apoptotic blebs (0.03–1 µm). For example, the terms apoptotic microparticles, small ApoVs, and even apoptotic bodies have been used to define the smaller apoptotic blebs (15, 40, 44, 45). For this reason, the term ApoVs will be used in this review to describe lipid encapsulated EV released from dying (apoptotic) cells.

Extracellular vesicles are becoming increasingly studied due to their release by cancer cells and their reported influence on the immune system, metastasis, angiogenesis, and coagulation (46–49). Although tumor-derived ApoVs are released in relative abundance following chemotherapeutic treatment, as compared to exosomes and MV (12, 47), limited research has been directed toward ApoV. Here, we will focus on the ApoV and their functional implications in cancer, the immune system, and coagulation.

## Cell Death: A General Overview

At present, at least six cellular processes leading to cellular death have been described: mitotic catastrophe, senescence, necrosis, necroptosis, autophagy, and apoptosis (50, 51). However, it is unclear whether the six cellular death mechanisms are strictly independent, or if they all eventually overlap to some degree.

During eukaryotic cell division (mitosis), DNA-damaging agents cause cells to lose or gain a single chromosome (an aneuploid state) that, if left unchecked, could lead to severe genomic instability (52, 53). This may result in irreversible damage and death of the aberrant dividing cell in a manner known as mitotic catastrophe (51, 53). Prior to the discovery of mitotic catastrophe, Hayflick et al. showed that normal cells eventually cease to divide *in vitro* despite the availability of favorable conditions for cell growth (54). This inflammatory death mechanism, defined as senescence, is now known to be triggered by several signals such as DNA damage or shortened/dysfunctional telomeres (38, 50, 51, 55, 56). Necrosis is the uncontrolled breakdown of the cell membrane and consequent release of intracellular contents and proinflammatory molecules into the extracellular matrix (57, 58). Several pathological conditions, such as infection, ischemia, or inflammation can cause necrosis and that is generally characterized by cellular swelling and organelle degradation (57, 59). Necrosis can be triggered in a controlled manner, a process known as necroptosis, and driven by receptor-interacting protein kinase 1, 3, and pseudokinase mixed lineage kinase domain-like (60, 61). Autophagy is triggered when redundant or unwanted proteins are excessively targeted for degradation by the cell’s proteolytic mechanisms (50). One of the main mediators of autophagy is ubiquitin, often leading to degradation within proteasomes (50, 62, 63). Apart from apoptosis, autophagy and necrosis are the only other types of cell death that are characterized by membrane blebbing (50, 58, 64).

Apoptosis is a highly controlled process and is activated *via* two main pathways: the extrinsic (or receptor) pathway is characterized by the binding of a ligand to a death receptor of a cell (65). Activation of these death receptors by their ligands may lead to the assembly of the Fas-associated death domain and caspase-8 (66). Apoptosis is orchestrated *via* the activation of a (usually inactive) cytoplasmic family of proteins known as caspases (67–69). The activation of one may lead to the activation of another and thus initiate apoptosis in a cascade fashion. Hence, within the extrinsic pathway, recruited, activated caspase-8 cleaves caspase-3 which will cleave other caspases, eventually leading to apoptosis (69, 70). The other pathway, known as the intrinsic pathway, also converges at caspase-3 (67). However, the intrinsic (or mitochondrial) pathway is usually triggered *via* stress signals that may lead the mitochondrion to the leakage of proapoptotic factors, including cytochrome c, into the cytoplasm (67, 69, 70). This results in the formation/activation of several protein complexes including caspase-9 which then cleaves caspase-3 leading to downstream disassembly of cellular components (70). One of the main features of apoptosis is the formation and release of membrane blebs or ApoV (71). One advantage of the apoptotic process is that proteins and nucleic acids, that would otherwise trigger an immune response, are packaged within these apoptotic blebs for rapid clearance by the immune system (72, 73). On the other hand, materials that act as autoantigens may also be packaged into apoptotic blebs (45).

## General Mechanisms of EV Formation

**Figure 1:**
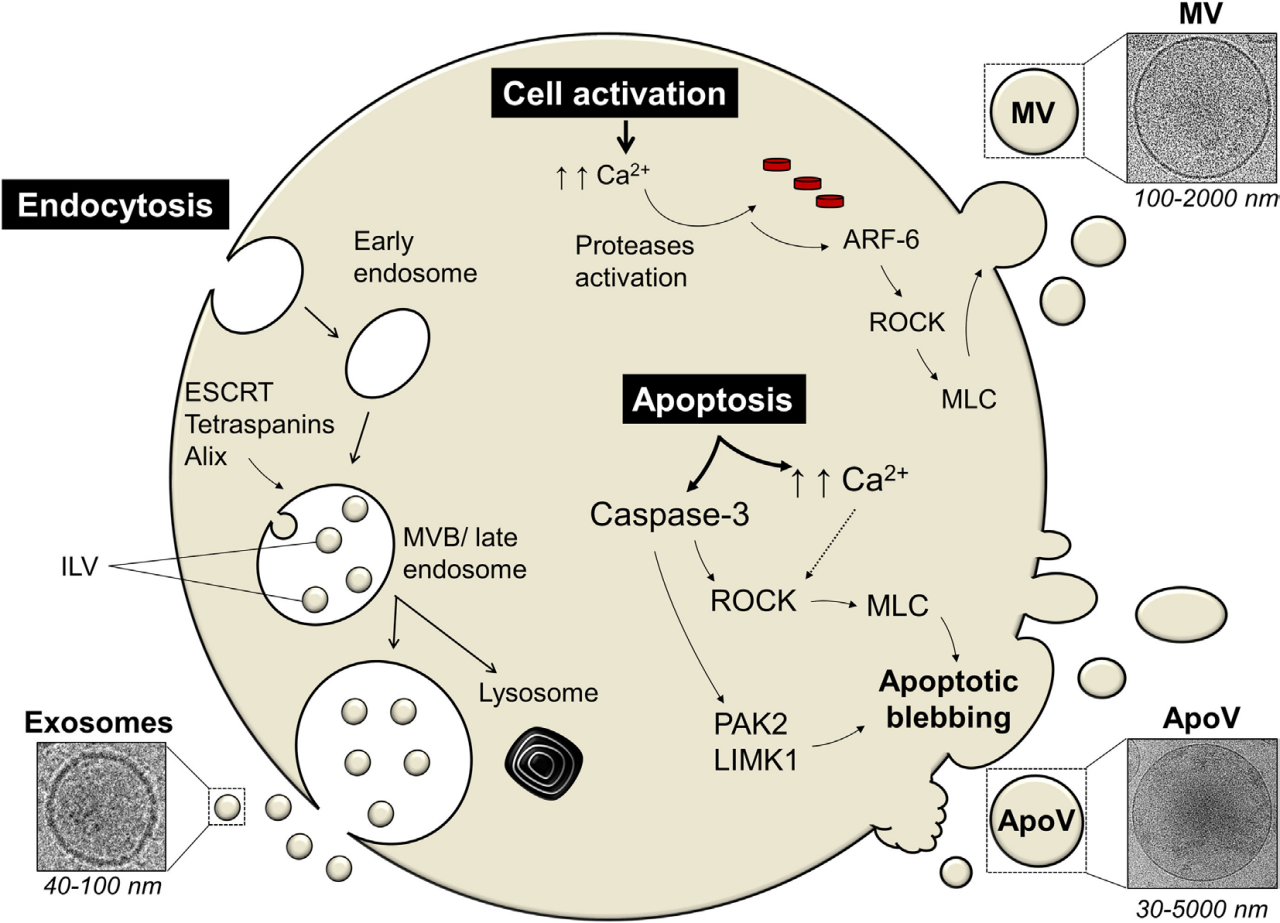
The mechanism of extracellular vesicles (EVs) release differs among different types of EV. Exosomes are secreted *via* the endosomal pathway, starting with the inward invagination of the cell membrane forming an early endosome. In later stages, further invagination of the endosome leads to the formation of intraluminal vesicles (ILV) and this late endosome is now referred to as a multivesicular body [MVB; Ref. (38, 76)]. The MVB can either be targeted for cellular destruction/recycling by lysosomes or it may fuse with the cell membrane thus releasing ILV as exosomes. Proteins involved in exosomal machinery include the endosomal sorting complexes required for transport (ESCRT), tetraspanins (such as CD9), ALG-2-interacting protein X (Alix), and tumor susceptibility gene 101 [TSG101; Ref. (74)]. Microvesicles (MV) and apoptotic vesicles (ApoV) are secreted *via* the direct outward budding of the cell membrane. Cellular activation or apoptosis results in an increased influx of calcium ions (Ca^2+^) which triggers proteases, such as calpain or gelsolin (80). These activated proteases lead to cytoskeletal disruption. ADP-ribosylation factor-6 (ARF-6) initiates a signaling cascade eventually activating the Rho-associated protein kinase (ROCK) signaling pathway which in turn activates myosin light-chain (MLC) kinases that activates and phosphorylates MLC that would initiate blebbing eventually leading to ApoV/MV release (81, 82). Apoptosis initiation also activates apoptotic enzymes known as caspases. Caspase-3 can cleave ROCK transforming it into a constitutive truncated form which then enhances the phosphorylation and activation of MLC that leads to ApoV shedding (83). Caspase-3-mediated blebbing can also act on other blebbing-mediated proteins, such as LIM domain kinase 1 (LIMK1) or p21-activated kinase (PAK2); reviewed in Ref. (71).

The mechanism of EV release is tightly regulated and differs between exosomes, MV, and ApoV (Figure 1). The exosomal machinery begins with the cell membrane invaginating inwards toward the intracellular matrix by endocytosis, forming an endosome (74). This early endosome is formed by the aid of proteins such as Ras-related in the brain GTPases and soluble *N*-ethylmaleimide-sensitive factor attachment protein receptor proteins (75). In later stages, further invagination of the endosome leads to the formation of intraluminal vesicles (ILV) and this late endosome is now referred to as a multivesicular body (MVB) (38, 76). Although their specific role remains unclear, tetraspanins, the endosomal sorting complexes required for transport (ESCRT) complexes, and ALG-2-interacting protein X, are involved in ILV formation (15). The excision of ILV into MVB requires ESCRT-III proteins, such as vacuolar protein sorting-associated protein 20, 24, and Snf7 (77). MVB can then either be targeted for degradation/recycling or may fuse with the cell membrane releasing ILV known as exosomes (15, 38, 74). Alternatively, lipid-metabolizing enzymes, such as neutral sphingomyelinase and phospholipase D2, can drive the formation of MVB and ILV in an ESCRT-independent manner (78, 79). However, it remains unknown how ESCRT and lipid-metabolizing enzymes cooperate to induce exosome formation (78).

Cells also release EV through the direct outward membrane budding and depending on the state of the cell, the EV is termed MV or ApoV. Initially, the shedding mechanism of MV or ApoV is thought to begin with the influx of calcium into activated or dying cells, respectively, resulting in the activation of calcium-dependent proteases, such as calpain and gelsolin (38, 80, 84, 85). This leads to the disruption of the membrane cytoskeleton, exposure of phosphatidylserine (PS), and initial formation of membrane protrusions (81, 85). ADP-ribosylation factor-6 initiates a signaling cascade eventually activating the Rho-associated protein kinase (ROCK) signaling pathway which in turn activates myosin light-chain (MLC) kinases that activates and phosphorylates MLC (38, 82, 86). The cell membrane then begins to bleb due to increased hydrostatic pressure following MLC-driven actomyosin contractions (87). Eventually ApoV or MV is released (81, 82). The mechanism of MV/ApoV scission remains unclear. However, it has been shown that vacuolar protein sorting four participates in the scission of T cell-derived MV (88). No incision proteins have yet been identified in ApoV release. One could speculate that the budding off of ApoV may resemble the mechanism of ILV excision. It has been hypothesized; however, that ApoV may simply break-off due to shear force under flow conditions and/or interaction of a recipient phagocyte “pinching” off ApoV (71).

Despite the mechanistic similarities between ApoV and MV, the apoptotic cell passes through a three-step apoptotic disassembly process to finally release ApoV [reviewed in Ref. (71)]. One major pathway of apoptosis is orchestrated *via* the activation of cytoplasmic caspases (67–69). Sequential activation of caspases leads to dismantling and repackaging of cellular and nuclear content caspase-3 (69, 70). The blebbing of the apoptotic cell marks the first step of apoptotic disassembly and is linked to caspase-3 (83, 89, 90). There is evidence that caspase-3 can cleave ROCK-1 transforming it into a constitutive truncated form which then enhances the phosphorylation and activation of MLC (89–91). Caspase-3 can also act on other blebbing-mediated proteins, such as LIM domain kinase 1 or p21-activated kinase (92, 93). The blebbing cell then moves to stage two whereby membrane protrusions form *via* microtubule spikes and/or long string-like constructs known as apoptopodia (94, 95). Finally, the apoptotic cell fragments, and the formed ApoV, detached from the membrane protrusions and are released.

## Molecular Profile of EV

The composition of EV largely depends on the type and differentiation state of the parental cell. Currently, differential ultracentrifugation is the most common technique to isolate EV. Exosomes are isolated using ≥100,000 × *g* along with pore filtration to remove larger EV fractions (21, 23). However, it has proved difficult to isolate the larger MV or ApoV, and to obtain a pure EV preparation as ApoV or MV purified from identical centrifugation speeds may cross-contaminate due to spontaneous cell death or MV release (47). Despite these limitations, various proteins, DNA, RNA, and lipid profiles have been identified to assist in the phenotyping of MV and ApoV (23, 47, 96–98). Current methodology for protein detection in EV includes western blotting, flow cytometry, and mass spectrometry (24, 47). It is now clear that many of the proteins enriched in EV are involved in EV formation or trafficking (3). Despite the different types of EV, there are well-documented markers that are commonly shared among EV fractions (24). Cluster of differentiation 147 (CD147), for example, is consistently observed to be enriched in tumor-derived EV (47, 99, 100). Acting as an Extracellular Matrix Metalloproteinase Inducer as a main role, CD147 is thus also referred to as “EMMPRIN.” Once matrix metalloproteinases (such as MMP1, MMP2, and MMP11) are induced by CD147, the enzymes help to break the extracellular matrix thus aiding in the proliferation of tumor cells (101–103). With a family of at least 30 proteins, tetraspanins levels are also elevated in EV (104–106). However, recent studies have shown the expression level of the tetraspanin CD9 is lower in ApoV compared to exosomes or MV (47, 96, 99, 107). Tetraspanins are glycoproteins suspected to be involved in cell motility, adhesion, and proliferation, and are known to complex with integrins (108, 109). It is, therefore, not surprising that adhesion molecules, such as integrins are also detected in EV (23, 47, 96, 99). Despite all the efforts, there has not been a stand-alone protein marker for ApoV that can distinguish them from other EV types (47, 96).

Interestingly, the DNA-associated proteins, histones, were assumed to be exclusive ApoV markers (46). Despite this, a proteomic study of dendritic cells (DC)-derived exosomes by Théry et al. detected histones in exosomes (15). They hypothesized that their presence may be due to the spontaneous DC apoptosis, contaminating the DC-derived exosome preparations with disintegrating nuclear material. However, it is now appreciated that histones localize within the cytoplasm, as well as the nucleus (110), and histones within exosomes and ApoV have been widely documented (45, 47, 111). Since EV contain nucleic acids, including RNA, it is likely that histones in EV act as chaperones for nucleic acids (46, 112). It has been proposed that the presence of RNA in exosomes may be due to the fact that MVB contain RNA-induced silencing complexes (113–115) where there is a direct interaction between histones and RNA (113). A study by Müller et al. suggests that the histone H3 modification is necessary for exosome release (116). Furthermore, glioma-derived EVs have been known to carry mRNA, micro RNA, and proteins that contribute to tumor growth (112, 117). The uptake of glioma-derived MV, containing oncogenic epidermal growth factor receptor, by endothelial cells have been known to greatly alter the nature of the endothelial cells in a manner that elevates tumor angiogenesis (117). However, a recent study revealed that there is a clear difference in the RNA profile between ApoV, MV, and exosomes, highlighting that ribosomal RNA and smaller RNA is highest in ApoV (118). Previous studies have identified that the RNA content in EV is reflective of the RNA content of the cell of origin (115, 119). Since DNA fragmentation is a feature of apoptosis, it was no surprise that DNA fragments have been detected in ApoV (120, 121). These DNA fragments can be transferred to recipient cells and reused when ApoVs are engulfed by phagocytes (121).

The exposure of PS on the outer leaflet of EV membranes is a common feature among EV (7, 21, 122). PS is a membrane aminophospholipid that is actively kept within the intracellular level (inner leaflet) of a living cell by the (predicted) ATP-dependent membrane lipid transporters known as flippases (123–125). Simultaneously, the other group of (predicted) lipid transporters, floppases, transports choline- and amino-phospholipids toward the outer membrane leaflet (126). The rate of floppase activity is usually 10 times slower compared to flippase (127). However, during apoptosis or cell activation, flippase is believed to be inhibited and different lipid transporters, scramblases, are activated (128–130). Unlike the other two lipid transporters, scramblases are calcium-dependent and ATP-independent (123, 131). The activity of scramblase transporters moves all major classes of phospholipids back and forth, thereby destroying the phospholipid asymmetry orchestrated by flippases and floppases (128). The presence of PS at the extracellular leaflet by scramblases is a key marker of apoptosis and is particularly enriched in ApoV (47, 96, 123).

Since EV originate from the cell membrane, they are expected to carry various cellular surface glycoproteins and glycolipids [commonly known as glycans; Ref. (132)]. Most studies have focused on exosomes for glycan profile, and demonstrate a complex and varying glycan content depending on the cell of origin (133–137). The use of plant-derived carbohydrate-binding proteins (lectins) and mass spectrometry has been an integral part of glycan profiling (136–139). Glycomic analysis remains challenging; however, due to the lack of specific glycan ligands and the fact that a large number of low molecular weight carbohydrate molecules are the building blocks of various molecular structures (140). Thus, the glycomic profiles of EV remain refractory to analysis and, therefore, a major hurdle to laboratories studying the molecular composition of EV. Nevertheless, studies have shown that the glycans detected in EV participate in protein trafficking, attachment, and EV uptake by recipient cells (134, 136, 141–144). The glycome of mammalian cells-derived EV shows consistency in terms of lectin interactions suggesting the presence of *N*-acetylglucosamine as an integral component of most EV glycans (140).

The surface of EV is negatively charged partially due to the heavy sialylation of surface glycans (47, 49, 138, 145–147). Increase in sialylation is also a common feature of tumor cells along with other proteins such as the superfamily tetraspanins (148, 149). It remains to be determined if this anionic-rich surface due to sialylation dictates the function of EV. Nevertheless, EVs have a sialic acid-rich surface and EV bind to the sialic-acid binding lectin CD169 (47, 49, 138). Interestingly, CD169 has a low (mM) affinity to sialic acids and thus only heavily sialylated structures (such as tumor-derived EV) are able to bind avidly to CD169^+^ macrophages (150). Moreover, CD169 binds to α2,6 and α2,3 sialic acids of EV but prefers the latter [discussed later in Ref. (47, 49)].

## ApoV and the Immune System

Tumor antigens can be captured by antigen-presenting cells (APC) *via* direct cell-to-cell contact with living or apoptotic cells (151), heat-shock-associated and soluble proteins, or tumor-EV (19, 152–155). Tumor antigens captured by APC activate CD4 helper and cytotoxic lymphocyte-driven immune responses for tumor regression (156–159). Previous reports indicate that EVs display major histocompatibility complex 1 (MHC-I) and MHC-II on their surface and theoretically should be capable of antigen-presenting function (4, 81, 160). However, cross-presentation of apoptotic cell and EV-associated antigens are more often observed *in vivo*, rather than direct antigen presentation by the vesicles themselves (19, 161–163). Dendritic cells (DCs) appear to be the major APC subset able to efficiently present antigens derived from apoptotic cells to stimulate both MHC-II and MHC-I-restricted CD4 and CD8 T cell responses (162). Exploiting the ability of DC-derived exosomes to eradicate established tumors due to the expression of MHC-I and -II on the surface of the exosomes (164) can be useful for exosome-based vaccine therapy. Heat-shock proteins have been detected in exosomes (23, 99), and have been reported by Srivastava’s group to participate in immunogenic action *via* their interaction with APC (165).

Paradoxically, studies regarding apoptotic cell-associated antigens have either identified them as immunosuppressive (166–168) or immunostimulatory (169–171), depending on the experimental setting. Recent studies suggest that tumor-derived ApoV can downregulate the immunostimulatory effect of antigen-specific DC *in vivo* (47, 167). There is evidence that the immunosuppressive effect of apoptotic cells and ApoV is caused by the transforming growth factor β1 [TGF-β1; Ref. (167, 172, 173)]. PS participates in the immunosuppression by ApoV through the induction of TGF-β from tissue-resident macrophages (174). Interestingly, the same study showed that TGF-β was not released when apoptotic cells failed to express PS (174). A recent study also highlights that the culture content of chemotherapy-treated tumor cells (floating dead cells, supernatant, and potentially ApoV) promotes primary tumor growth (175). The study also showed that this tumorigenic effect was caused by macrophages releasing proinflammatory cytokines known to promote tumor growth, and was PS-dependent (175).

In contrast, exposure of DC to murine myeloid cell-derived ApoV resulted in DC maturation and the secretion of proinflammatory cytokines (176). Leukemic-derived ApoV can elicit DC-driven CD8 T cell activation (177) and the immunization of antigen-pulsed tumor-derived ApoV alone elicits a significant CD8-mediated and anti-cancer immune response in mice (47). In addition, compared to MV or exosomes, tumor-derived ApoV afforded the highest anti-tumor protection against a specific antigen, *via* unknown mechanisms (99). Intriguingly, ApoV elicited the highest protection despite containing the lowest level of tumor antigen (ovalbumin), as compared to MV and exosomes (99). Despite its documented role in immunosuppression (174, 175), PS may also act as an immune stimulant and, therefore, it is possible that PS enrichment on the surface of ApoV could be responsible for their superior immunogenic activity. Hoffman et al. identified that apoptotic cell clearance is enhanced by PS-receptor mediated micropinocytosis [a regulated form of endocytosis for solute molecules and antigens; Ref. (178, 179)]. The pathway is highly active among APC such as macrophages and DC (179). Moreover, it was shown that blocking PS on MV (by annexin V) disables their uptake by target cells (29).

For apoptotic cells and their ApoV to be efficiently cleared by phagocytes, their exposed PS functions as an “eat-me” signal to phagocytes (71, 178). First, PS binds to annexin V which in turn is recognized by phagocytes (123, 180). However, PS is also a known ligand for other receptors, such as β2-glycoprotein I (181), Mer receptor tyrosine kinase (182), lectin-like oxidized low density lipoprotein-receptor 1 (183), and PS-receptor (184), all of which are known to promote apoptotic cell clearance (182, 184–186). For example, PS-receptor-deficient mice died due to accumulation of uncleared apoptotic cells in lung alveoli (184). A sufficient threshold of PS exposure is necessary for phagocytic clearance. Borisenko et al. have measured phagocytosis of live, apoptotic, and live with the inclusion of exogenous PS (187). Their study concluded that phagocytosis is directly proportional to PS levels. Thus, PS exposure is a critical factor for the clearance of apoptotic materials. Although PS and its receptors are critical for phagocytic recognition, the Albert group have demonstrated that integrin subunits α_V_β3 and α_V_β5 on macrophages and DCs, respectively, participate in phagocytic clearance of apoptotic cells, but these studies did not investigate a possible involvement of PS in the phagocytic process (162, 188). Furthermore, oxidation levels on apoptotic cells and ApoV create a binding site for thrombospondin and the complement protein C3b, which are in turn recognized by phagocytes (38, 189, 190). CD44-deficiency has been shown to impair apoptotic cell clearance in the lungs (191). This coincides with our study that showed that ApoV express higher CD44 than MV and exosomes (99). The fact that apoptotic cells and their ApoVs are efficiently recognized by the immune system opens a potential use of ApoV for vaccination against cancer. Indeed, PS exposure is seen in all EV (38, 46, 99, 192), and PS participates in the uptake of exogenous antigens (29, 193). However, the slight increase of PS levels on ApoV (96, 99), above that observed for MV and exosomes could still be the threshold necessary to induce a superior immune response. There have been several clinical trials for cancer treatments using exosome-borne tumor antigens, but with poor outcome (194–197). Nevertheless, since immune cells efficiently recognize antigens on ApoV (47, 170, 177, 198), ApoVs remain of interest for cancer immunotherapy.

ApoV can act as immunostimulatory or immunosuppressive. This greatly highlights our lack of understanding of the complexity of ApoV interactions with the immune system. Adding to the complexity of our understanding, *in vitro-*generated ApoV is not immediately engulfed, as would be expected to happen *in vivo*. Therefore, the results of *in vitro* studies may not always reflect the true *in vivo* situation. Unfortunately, there are limited studies regarding tumor-derived ApoV and their implications for cancer and the immune system. Because tumor-derived ApoVs are strongly thrombotic (see below), this makes it difficult to conduct immune activation studies utilizing systemic application of ApoV regarding of tumor-derived ApoV (48, 99).

## Could CD169 be the Gate for EV-Driven Lymph Node Metastasis?

One of the most important events of tumor metastasis is the migration of tumor cells from the primary site to the draining lymph node [LN; Ref. (199)]. Invading tumor cells eventually spread to other LNs in a sequential fashion starting and ending from the closest (draining) to most distal LN, respectively (200). In fact, there is convincing evidence that draining LNs are the best prognostic estimators for the status of the entire lymphatic nodal system (201). Within the subcapsular sinus (SCS) of LN, macrophages are the first to be exposed to antigens and have a role in presenting the captured antigens to APC, including B cells (202). Hood et al. have demonstrated that B16F10-derived exosomes accumulate in and prepare the draining lymph node for tumor invasion (203). This mechanism is often termed “seed and soil” in metastasis, where tumor-derived EVs are regarded as “seeds” preparing a particular site (the soil) for tumor cell invasion (204, 205). The studies suggest that tumor-derived EVs facilitate invasion by enhancing angiogenesis and immunosuppression *in situ* (22, 203, 206). Interestingly, a macrophage-restricted receptor known as sialoadhesin (CD169; Siglec-1) is abundantly expressed on the surface of macrophages within the SCS of LN, marginal zones of the spleen, and liver [Kupffer cells; Ref. (49)]. CD169 is a member of the sialic-acid binding Ig-like lectin family of proteins; this enriched level of CD169 expression allows these CD169^+^ macrophages to bind to glycoproteins bearing terminal sialic acids (207). Depending on the experimental setting, CD169^+^ macrophages mediate a tolerogenic or immunogenic response to self-antigens, infection, and tumor models (208–212).

Several studies that deplete the entire CD169^+^ macrophage population (using diphtheria toxin or clodronate liposomes) indicate that the function of CD169^+^ macrophages is immunogenic (213–216). In cancer, the Tanaka group’s CD169^+^ macrophage depletion model showed that the cells have a critical role in the anti-cancer effect (212). Interestingly, they show that upon immunization with dead tumor cells, CD169^+^ macrophages cross-present tumor antigens to CD8^+^ T cells thereby mediating a cytotoxic-mediated anti-cancer immune response. Consistent with these findings, Pucci et al. showed that tumor spread is significantly reduced when tumor-derived EV are captured by CD169 (209). The results showed that CD169 poses a physical barrier to block tumor-derived EV interactions with LN B cells preventing pro-metastatic humoral immunity (209).

**Figure 2:**
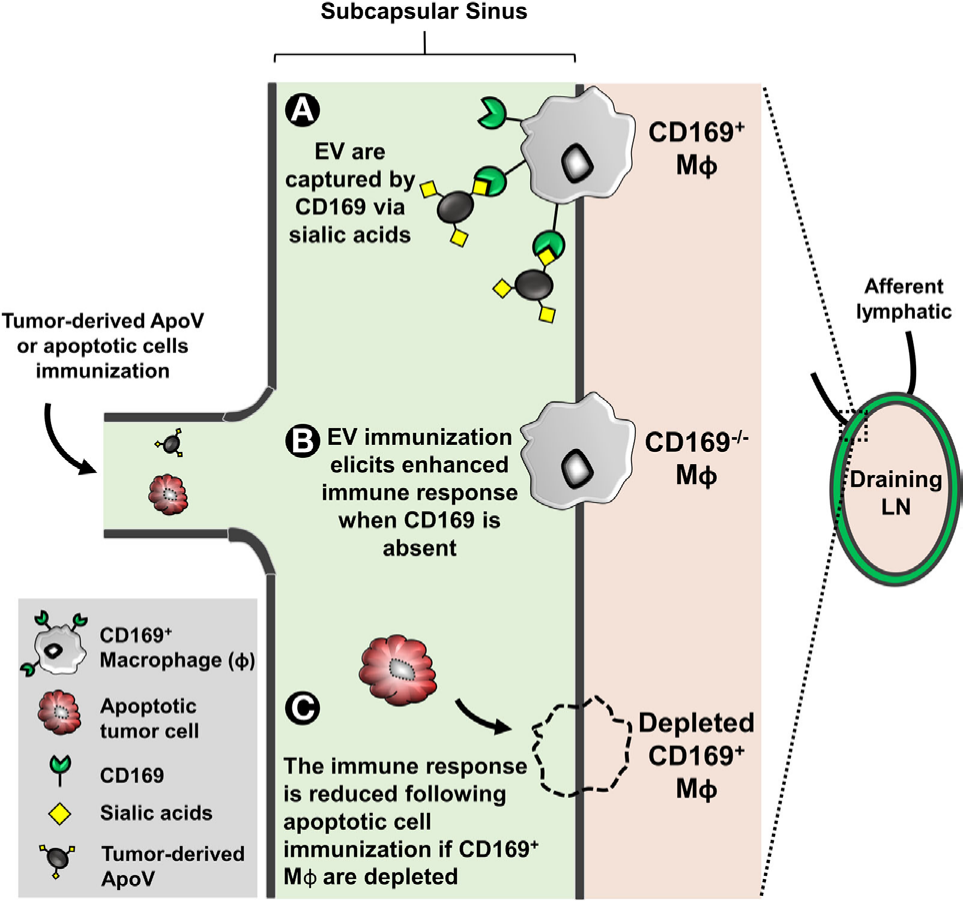
The subcapsular sinus of lymph nodes (LN) is rich in macrophages expressing the surface molecule CD169. EVs are enriched in surface sialylated ligands. **(A)** CD169^+^ macrophages can bind to these EV in a sialic acid-dependent manner. CD169 binds to α2,6 and α2,3 sialic acids but prefers the latter (47, 49). **(B)** EL4 (thymoma)-derived apoptotic vesicle (ApoV) immunization, CD169^−/−^ mice elicit a significant enhanced cytotoxic response (47). The invasion of tumor cells from the primary site to the draining LN is unaffected by CD169 (217). However, the direct implication of EV and tumor metastasis with respect to CD169 remains unknown. **(C)** When depleted, CD169^+^ macrophages have a critical role in the anti-cancer effect (212). CD169^+^ macrophages cross-present tumor antigens from dead tumor cells to CD8^+^ T cells thereby mediating a cytotoxic-mediated anti-cancer immune response.

The function of the high surface sialylated state of EV remains unclear, however, studies have identified that CD169^+^ macrophages exclusively capture EV in a CD169-sialic acid-dependent manner (47, 49). EV-immunized CD169-deficient mice display a significant elevated immunogenicity, suggesting that the function of the CD169 receptor itself may be immunoinhibitory [Figure 2; Ref. (47, 49)]. However, our recent study suggested that progression of primary tumor growth and LN metastasis was not significantly associated with CD169 expression in mice (217). Despite this, the direct implication of CD169 capture of EV (the “seed”) and CD169 (the “soil” receptor) in tumor metastasis has not been extensively investigated. Since cancer patients have an increased level of circulating tumor-derived EV bearing pro-metastatic factors highlights the urgency to further explore the role of the tumor EV receptor CD169 in cancer progression.

## Tumor-Derived ApoV and Coagulation

Thrombosis is a pathophysiological condition characterized by localized clotting of the blood within a blood vessel leading to a blockage of blood flow (218, 219). In venous thromboembolism (VTE), the wall of the endothelium remains intact but may transform from an anticoagulant to a procoagulant surface (218, 219). Cancer patients have a fourfold increased risk of developing VTE (220, 221). Strikingly, this risk is increased to more than sixfold when the patients are receiving chemotherapy (221, 222). The risk of VTE depends on the cancer type and stage as well as the type of anti-cancer drug administered, which may alter the hemostatic state in patients (223, 224). Tamoxifen, gemcitabine, and platinum-based compounds, for example, are known to lower the levels of circulating anticoagulants (225–228). In contrast, thalidomide treatment for leukemia does not increase the risk of VTE unless combined with other drugs (229, 230). In general, solid tumors pose a greater risk of VTE and worsened prognosis as compared to lymphomas (231). It is now evident that the leading cause of death in cancer patients receiving chemotherapy is VTE (223, 224).

The shedding of procoagulant EV from human cancer cells was first reported in 1981 (232). In the later decades, tissue factor (TF; CD142) and PS exposure were identified as the main procoagulant components of EV (233). TF expression in tumor cells is linked to the mutations in p53 and phosphatase and tensin homolog PTEN (234), resulting in dysregulation of TF expression which may be upregulated 10^5^-fold compared to non-malignant counterparts (235). TF is an integral membrane protein with a MW of approximately 45 kDa. TF is not only present on the surface of most non-hematopoietic tumors but is also present in EVs released from these tumor cells (47, 48, 99, 235–238). Although the majority of procoagulant TF is vesicle associated, an alternatively spliced soluble form of TF acts independently of FVII to stimulate angiogenesis (239, 240). Moreover, the cytoplasmic domain of TF is involved in signaling events that promote tumor metastasis, further demonstrating that TF displays pro-metastatic function independent of FVII (241).

**Figure 3:**
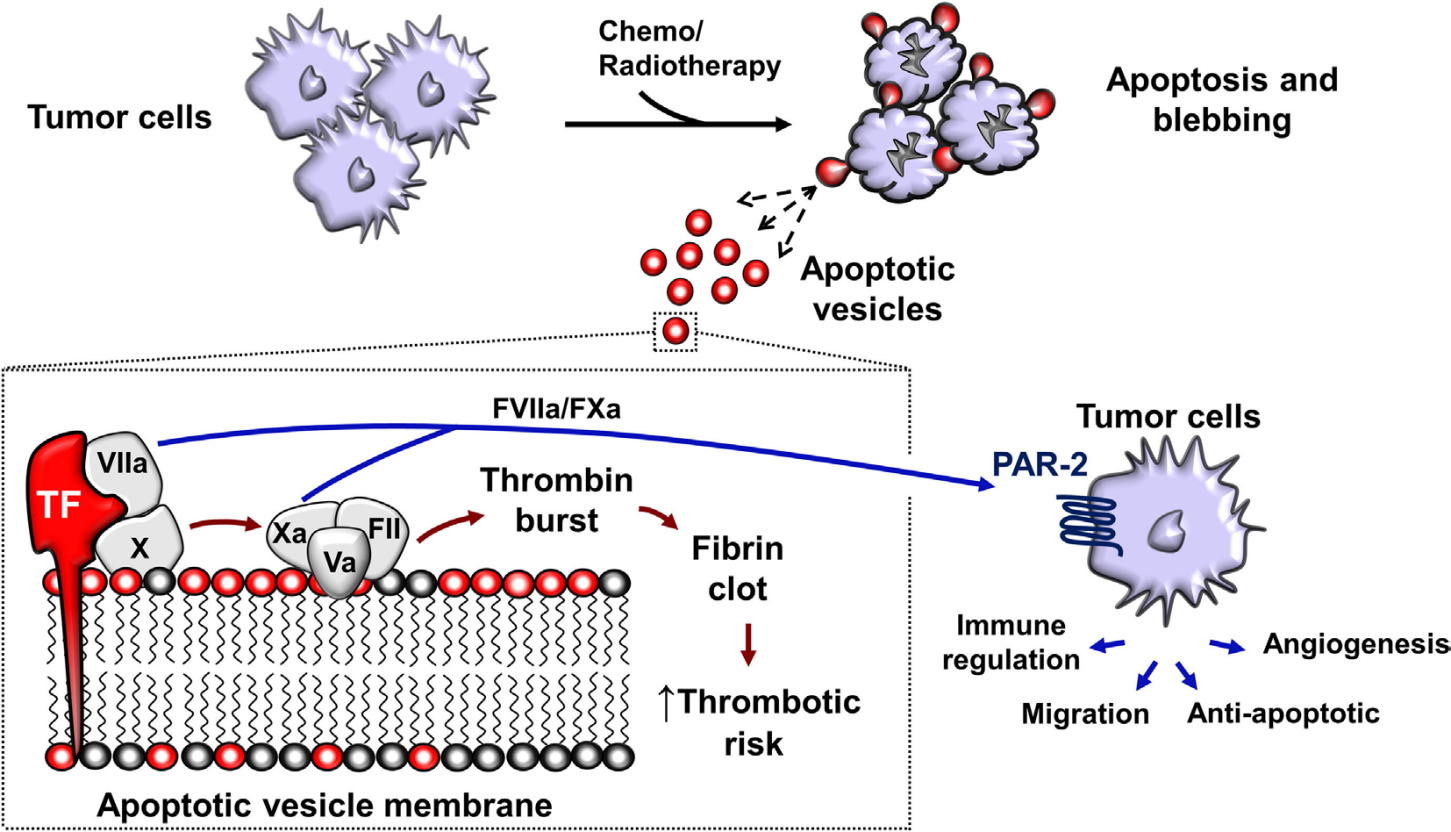
Chemotherapy or radiotherapy-exposed tumor cells may initiate apoptosis and trigger the release apoptotic vesicles (ApoV). Tumor-derived ApoVs express tissue factor (TF) and harbor an anionic-rich surface due to phosphatidylserine (PS) exposure. TF/PS procoagulant complex binds with activated factor VII (FVIIa) and activates the extrinsic coagulation cascade that eventually leads to a fibrin clot. *In vitro*, tumor-derived ApoV are significantly more procoagulant than MV, exosomes, or their cell of origin (47, 48, 99). FVIIa and FXa also cleaves protease-activated receptor-2 (PAR-2) present on the surface of tumor cells (250–252). This results in signal transduction events important in angiogenesis that enhances tumor blood supply and tumor cell growth (234). ApoVs are, therefore, a potential source of increasing the risk of thrombosis and metastasis in cancer patients undergoing chemotherapy.

The association of tumor-derived EV and thrombotic risk is now well-appreciated (193, 233, 242–244). Cancer patients have a significantly higher number of circulating EV compared to healthy controls (243–245). For example, pancreatic cancer patients undergoing chemotherapy are known to possess elevated levels of TF-bearing EV, thus increasing the risk of VTE (246). This phenomenon was observed by Zwicker et al. when they concluded that TF-bearing EVs were associated with VTE in cancer patients (244). Since VTE risk is increased by chemotherapy, this implicates tumor-derived ApoV released from dying tumor cells (Figure 3). Although the levels of detected EV in chemotherapy patients are increased, it remains difficult to determine their identity as either tumor, leukocyte, platelet, or endothelial-derived particles. In addition, there is a vast overlap between tumor-derived exosomes, MV, and ApoV with respect to their size, lipid compositions, surface markers, morphology, and functional behavior (21, 96, 99). *In vitro* assays indicate that tumor-derived ApoV are significantly procoagulant in a TF/PS-dependent manner (48, 99, 247). The predisposition to VTE observed in cancer patients may be due to the close association of tumor with the extensive networks of vasculature, allowing the direct interaction of tumor-derived EV with blood-borne coagulation factors. However, the depth of the contribution of local release of tumor EV to the systemic hypercoagulable state observed in cancer patients remains to be elucidated (220, 222, 248, 249).

The presence of TF on ApoV may contribute to metastasis, since TF-mediated coagulation aids cancer progression (241, 253). In 1995, Bromberg et al. were the first to demonstrate a coagulation-independent role for metastasis by TF through mutation of a TF region required for the initiation of coagulation (241). Interestingly, despite the dramatically lower ability of the extracellular mutant to initiate coagulation, metastasis still occurred. This coagulation-independent effect was likely due to the additional function of TF in the activation of the protease-activated receptor-2 (PAR-2), present on the surface of tumor cells (250–252). TF binds to and activates factor VII, activated factor VII (FVIIa) cleaves PAR-2, resulting in signal transduction events important in angiogenesis that enhances tumor blood supply and tumor cell growth (234). Later studies suggested that the formation of the TF:FVIIa complex was necessary to induce metastasis *via* the inhibition of apoptosis, promoting cell adhesion, and angiogenesis (254–256). TF expression on tumor cells has been widely implicated in triggering thrombotic events in cancer patients (236). With the possibility that metastatic tumor cells may upregulate TF up to ~1,000-fold compared to non-metastatic cells (235), TF can be considered as a potent pro-metastatic molecule present on solid and myeloid leukemia-derived ApoV (47, 48).

## Conclusion

Despite the current advances in the field, much remains to be identified about ApoV and their composition, immune clearance, and their implications in cancer and coagulation. Furthermore, it remains unclear if tumor-derived ApoV generated by varying apoptotic agents possess distinct molecular, or morphological, or functional differences. There is greater need to improve on the isolation methods of EV to enhance their purity and decrease any co-contamination between different subtypes. Since ApoV suppress or stimulate an immune response, then they can potentially be used to treat autoimmunity or cancer, respectively. As for the latter, the immunogenic nature of ApoV opens the possibility to exploit their molecular composition for clinical utility as prophylactic and therapeutic cancer vaccines.

## Author Contributions

MM-S wrote the bulk of the review and made the figures. AM contributed to planning, scientific input, and editing of the manuscript.

## Conflict of Interest Statement

The authors declare that the research was conducted in the absence of any commercial or financial relationships that could be construed as a potential conflict of interest. The reviewer RX and handling Editor declared their shared affiliation.
